# High-altitude cerebral edema and high-altitude pulmonary edema in a trekker with a decent ascent profile: a case report

**DOI:** 10.1097/MS9.0000000000005047

**Published:** 2026-04-23

**Authors:** Suraj Bhatta, Surya Prakash Joshi, Suraj Shrestha, Ranjeet Ghimire, Madhav Raj Karki, Sanyukta Gurung, Sanjeeb Bhandari

**Affiliations:** a Himalayan Rescue Association, Kathmandu, Nepal; b Mountain Medicine Society of Nepal, Kathmandu, Nepal; c Maharajgunj Medical Campus, Institute of Medicine, Maharajgunj, Kathmandu, Nepal; d Birat Medical College and Teaching Hospital, Biratnagar, Nepal

**Keywords:** acclimatization, altitude sickness, cerebral edema, high-altitude, pulmonary edema

## Abstract

**Introduction and importance:**

High-altitude illness (HAI) comprises a spectrum of disorders, including acute mountain sickness (AMS), high-altitude pulmonary edema (HAPE), and high-altitude cerebral edema (HACE). Although gradual ascent is the most effective preventive strategy, severe complications such as HAPE and HACE can still occur in susceptible individuals despite recommended ascent profiles.

**Case presentation:**

A 31-year-old male trekker in the Manang Valley (3350 m) developed HAPE and HACE despite gradual ascent and 6 days of acclimatization. He presented with severe headache, nausea, and shortness of breath. Examination showed cyanosis, SpO_2_ 39%, respiratory rate 35, bilateral lung crackles, and positive cerebellar signs. Prompt treatment with oxygen, dexamethasone, acetazolamide, and nifedipine led to improvement. He was airlifted to a lower altitude and fully recovered.

**Clinical discussion:**

Despite a gradual ascent and absence of comorbidities, the patient developed concurrent HAPE and HACE, illustrating that severe illness can occur even in low-risk profiles. This underscores the role of individual susceptibility and highlights the importance of maintaining a high index of suspicion in all high-altitude trekkers.

**Conclusion:**

This case illustrates that even with proper acclimatization and ascent, severe forms of HAI, such as HAPE and HACE, can occur. It underscores the role of individual vulnerability, possibly influenced by genetic and environmental factors, in the pathogenesis of altitude illness. Prompt recognition and early intervention are essential to prevent fatal outcomes.

## Introduction and importance

High-altitude illness (HAI) encompasses acute mountain sickness (AMS), high-altitude cerebral edema (HACE), and high-altitude pulmonary edema (HAPE), all of which are associated with an ascent to elevations of 8000 feet (≈2500 m) or higher above sea level^[^1,2^]^. HACE represents the severe end of the spectrum, characterized by ataxia and altered mental status, and can progress to coma or death if not promptly treated. HAPE, on the other hand, manifests with exertional dyspnea, fatigue, dry cough, and cyanosis and may progress to the expectoration of pink, frothy sputum[3].

HIGHLIGHTS

- High-altitude illness can occur in healthy individuals despite gradual ascent and no prior altitude exposure.
- Early recognition, prompt pharmacologic therapy, and evacuation led to complete recovery of high-altitude illness without long-term complications.
- Risk factors beyond the rate of ascent, such as individual susceptibility, genetic predisposition, or environmental stressors, play a crucial role in the development of altitude illness.

HAPE typically develops 2–5 days after rapid ascent to altitudes above 2500–3000 m. It is uncommon below this threshold or after a prolonged stay at a given altitude[4]. According to a study by Richalet *et al*, ascending at a rate greater than 400 m per day is strongly linked to severe altitude illness. A gradual ascent allows for physiological adaptation and reduces the incidence of AMS and its complications^[^5–7^]^.

In a Swiss study involving 827 climbers, individuals with a prior history of AMS had a 58% risk of recurrence with rapid ascent and no pre-acclimatization, compared to only 7% with slow ascent and prior exposure. Among those without prior AMS history, 31% developed AMS with rapid ascent and no pre-exposure, compared with only 4% with slow ascent and acclimatization[8]. Similarly, in individuals with no known history of HAPE, the incidence was 0.2% when ascending to 4500 m over 4 days, but increased to 6% when the same altitude was reached within 1–2 days[9]. While ascent rate, maximum altitude, and pre-acclimatization are key risk factors, genetic predisposition may explain why some individuals develop HAI despite following recommended guidelines^[^9,10^]^. This manuscript complies with the TITAN Guidelines 2025 for the responsible and transparent use of artificial intelligence in academic publishing[11].

Following CAse REport (CARE) guidelines, we report here a case of HACE and HAPE in a 31-year-old male trekker who presented with headache, nausea, and shortness of breath during his ascent to Manang[12].

## Case presentation

A 31-year-old physically active male from the Czech Republic, with no known prior comorbidities, presented to the aid post (3540 m) with a 2-day history of headache accompanied by nausea and a 1-day history of shortness of breath while trekking. The headache was sudden in onset, severe in intensity, and associated with nausea but no vomiting. The dyspnea was acute in onset, persistent, and present at rest (mMRC Grade 4) and was accompanied by a productive cough with blood-tinged sputum. He further reported mild-moderate fatigue and dizziness on ambulation. He had no prior history of high-altitude exposure.

The patient’s trekking history revealed a gradual ascent from Syange to Yak Kharka over 6 days, with appropriate acclimatization stops. He had not taken any prophylactic medications such as acetazolamide. There was no history of preexisting cardiovascular disease or prior episodes of altitude illness.

**Figure 1.**
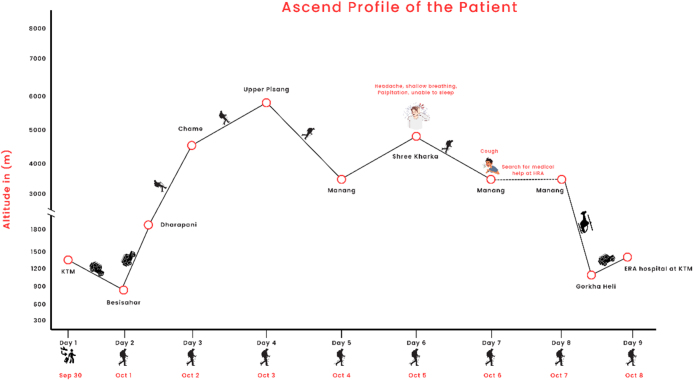
Ascent profile of the patient over 7 days.

The trekker followed a gradual ascent profile over 7 days on the Annapurna Circuit. He initially traveled from Kathmandu to Besisahar (760 m) by jeep, then continued by jeep to Dharapani, and hiked to Chame (2710 m) on Day 2. On Day 3, he trekked to Upper Pisang (3300 m), and on Day 4, he ascended to Manang (3540 m) without symptoms. On Day 5, he continued ascending to Shree Kharka (4040 m), where he developed a headache, shallow breathing, tachycardia, and sleep disturbance. Due to worsening symptoms, he descended back to Manang (3540 m) the following day, representing a descent of approximately 500 m. Symptoms persisted, and he presented to the Himalayan Rescue Association clinic in Manang on Day 7 for medical evaluation. Overall, the patient spent approximately 4 days above 3000 m, including 2 days above 3500 m, before seeking medical care. A detailed ascent profile is provided in Table 1 and illustrated in Figure 1.

**Table 1:** Ascent profile of the patient over 7 days.

Day	Route/activity	Altitude (m)	Symptoms
1	Kathmandu → Besisahar (by jeep)	760	None
2	Besisahar → Dharapani (by jeep), then hiked to Chame	2710	None
3	Chame → Upper Pisang (hike)	3300	None
4	Upper Pisang → Manang (hike)	3540	None
5	Manang → Shree Kharka (hike)	4040	Headache, shallow breathing, high heart rate, and sleeplessness (~9 PM)
6	Shree Kharka → Manang (descent)	3540	Symptoms persisted; developed a cough in the afternoon
7	Stayed in Manang; visited HRA clinic	3540	Medical evaluation for ongoing symptoms

**Figure 2.**
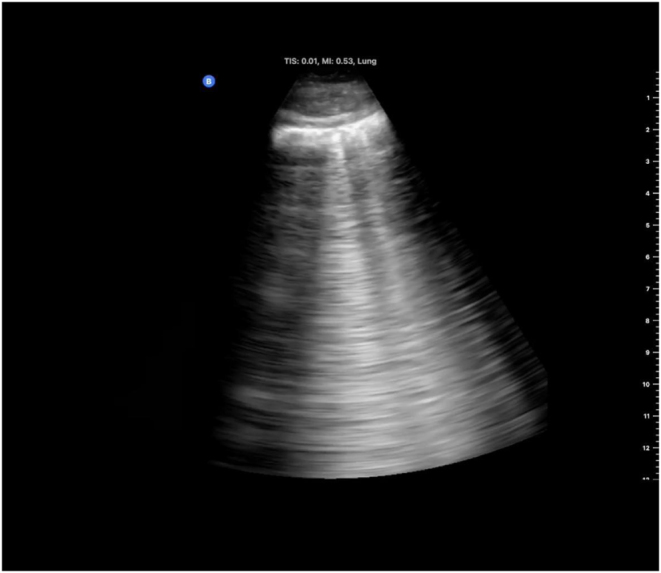
Portable chest ultrasonography showing multiple B-lines.

On examination, the patient was alert, cooperative, and well-oriented but appeared pale, with cyanosed lips, profuse sweating, and increased work of breathing. He was afebrile, with a respiratory rate of 35 breaths per minute, a heart rate of 109 beats per minute, a blood pressure of 150/80 mmHg, and an oxygen saturation (SpO₂) of 39% on room air. Chest auscultation revealed equal air entry with bilateral crackles in the lower lung zones. Bedside chest ultrasonography showed multiple B-lines in the middle and lower lung fields (Fig. 2). Neurologically, his higher mental functions were intact; however, tandem gait and finger-nose tests were positive, suggesting early HACE.

A clinical diagnosis of HAPE with HACE was made based on symptoms, physical findings, severe hypoxemia, and neurologic signs.

Immediate treatment was initiated, including intramuscular dexamethasone (8 mg), oral acetazolamide (250 mg), sustained-release nifedipine (20 mg), and oxygen therapy at 8 L/min via an oxygen concentrator. Following treatment, the patient’s SpO₂ improved to 90% on room air, with a significant reduction in cyanosis and respiratory distress.

He was airlifted to Gorkha and subsequently transferred by road to Kathmandu, where he was admitted to a tertiary care hospital. Laboratory investigations, including complete blood count and serum biochemistry, were within normal limits. A chest X-ray (posteroanterior view) showed bilateral patchy infiltrates. Transthoracic echocardiography revealed an elevated pulmonary artery pressure of 52 mm Hg. Sustained-release nifedipine 20 mg was given three times over a 24-h period. Overnight, his SpO₂ dropped below 93%, necessitating supplemental oxygen. Over the following days, his symptoms gradually improved, although a mild cough persisted.

After 3 days of inpatient care, the patient was discharged with no residual symptoms except for a slight cough. A follow-up chest X-ray performed 1 week after hospital admission showed complete resolution of the infiltrates.

## Clinical discussion

Each year, thousands of travelers visit high-altitude locations in Nepal for trekking and cultural experiences, putting them at risk of AMS. This is a significant public health concern with notable economic impacts, especially in a country where tourism is the primary source of income. Manang village is in the northern part of the Manang district. It is situated at a height of 3350 m on the Annapurna Circuit. Previously, the trek from Besisahar (760 m) to Manang required a week. Nowadays, it is only a 7-h drive due to the availability of new roads and vehicles since 2014 AD. Cheaper road access and easy availability of vehicles for travel mean that many locals and tourists are at risk of developing altitude sickness due to rapid ascent without adequate time for acclimatization[13].

Risk factors for HAI include rapid ascent, residence below 900 m, exertion, a history of altitude illness, and certain preexisting cardiopulmonary conditions[14]. The patient, a resident of the Czech Republic with no prior high-altitude exposure or preexisting cardiopulmonary conditions, had followed a proper ascent profile but still presented with HAI symptoms at Manang aid post. Although gradual ascent and acclimatization are effective in reducing the risk of altitude illness, they do not eliminate it. Individuals may still develop AMS, HAPE, or HACE despite following recommended preventive measures[15]. This suggests that factors beyond the rate of ascent, such as individual susceptibility, genetic predisposition, or environmental stressors, play a crucial role in the development of altitude illness.

A similar case with a decent ascent profile in a 55-year-old South African female with no prior comorbidities was reported in 2014, who was trekking in the Khumbu region of Nepal[16]. However, in contrast to this study, she had symptoms of upper respiratory tract infection. Previous studies have shown that individuals with no prior history of high-altitude exposure may exhibit variable responses to hypoxia, leading to increased pulmonary vascular pressures, capillary leakage, and fluid accumulation in the lungs and brain[17]. The patient also did not have a history of high-altitude exposure, which may have contributed to his susceptibility to severe altitude illness. Diverse interactions between genetic factors and the environment most likely explain individual susceptibility or relative resistance to these hypoxia-induced illnesses. High-altitude residents from different regions exhibit unique physiological responses to hypoxia contributed by acquired or genetic adaptation[18]. Tibetans exhibit better hypoxic ventilatory response (HVR) and resting ventilation, whereas the Andean population shows a higher hemoglobin concentration and oxygen saturation[19]. Hypoxia-inducible factors induce the expression of genes that respond to hypoxia, which further explains genetic variations in hypoxia-associated illness[20].

Hypoxia and low partial pressure activate neurohumoral and hemodynamic responses, leading to excessive perfusion of vascular beds, increased hydrostatic capillary pressure, and capillary leakage, consequently leading to edema of the brain and lungs, which presents as signs of HAI^[^21,22^]^. Individuals with a reduced HVR, resulting in limited compensatory hyperventilation during low-oxygen conditions, are especially prone to developing AMS and HACE. HACE, in particular, is frequently associated with an increased susceptibility to vasogenic brain swelling and disruption of the blood–brain barrier resulting from hypoxia^[^23,24^]^. Conversely, HAPE tends to occur in individuals who exhibit an excessive pulmonary vasoconstrictive response to hypoxia, resulting in increased pulmonary arterial pressure and the development of non-cardiogenic fluid accumulation in the lungs[25]. Similarly, in this case, the development of HAPE and HACE despite a gradual ascent and appropriate acclimatization may be attributed to individual susceptibility, possibly involving an exaggerated hypoxic pulmonary vasoconstrictive response and a reduced HVR, along with unrecognized genetic predisposition to hypoxia-related vascular permeability.

The mainstay of treatment for HAPE and HACE is immediate descent to a lower altitude with supplementary oxygen or hyperbaric therapy. Acetazolamide and dexamethasone are frequently used as the treatment plan for AMS and HACE. Calcium channel blockers (e.g., nifedipine) are preferred for the acute management of HAPE^[^26,27^]^. Similarly, in our patient, as part of the treatment plan, we administered dexamethasone, acetazolamide, nifedipine, and oxygen therapy via an oxygen concentrator until evacuation was possible. Following treatment, the patient’s SpO_2_ improved, and his respiratory distress lessened significantly. He was then evacuated to a lower altitude and subsequently admitted to a hospital for further management, which rapidly relieved all of his symptoms.

Most cases of HAI reported in the literature are associated with rapid ascent or inadequate acclimatization. However, this case documents the occurrence of concurrent HAPE and HACE in a trekker who followed a relatively gradual ascent profile. Such cases highlight that recommended ascent guidelines significantly reduce risk but do not eliminate the possibility of severe altitude illness.

## Conclusion

While gradual ascent reduces the risk of altitude illness, individual susceptibility can still lead to severe outcomes like HAPE and HACE. Early diagnosis, prompt treatment, and awareness of altitude-related risks are essential for preventing serious complications and fatalities in high-altitude environments.

## Data Availability

All data and materials used in this editorial are derived from publicly accessible sources, including peer-reviewed articles and referenced scientific literature. Full citations are provided within the text to ensure transparency and facilitate further exploration.
